# Persistent, Long-term Cerebral White Matter Changes after Sports-Related Repetitive Head Impacts

**DOI:** 10.1371/journal.pone.0094734

**Published:** 2014-04-16

**Authors:** Jeffrey J. Bazarian, Tong Zhu, Jianhui Zhong, Damir Janigro, Eric Rozen, Andrew Roberts, Hannah Javien, Kian Merchant-Borna, Beau Abar, Eric G. Blackman

**Affiliations:** 1 Emergency Medicine, University of Rochester School of Medicine and Dentistry, Rochester, New York, United States of America; 2 Imaging Sciences, University of Rochester School of Medicine and Dentistry, Rochester, New York, United States of America; 3 Imaging Sciences, Biomedical Engineering, and Physics, University of Rochester, Rochester, New York, United States of America; 4 Cleveland Clinic Lerner Research Institute, Cleveland, Ohio, United States of America; 5 Athletics and Recreation, University of Rochester, Rochester, New York, United States of America; 6 University of Rochester, Rochester, New York, United States of America; 7 Hamilton College, Clinton, New York, United States of America; 8 Physics and Astronomy, University of Rochester, Rochester, New York, United States of America; University of Montreal, Canada

## Abstract

**Introduction:**

Repetitive head impacts (RHI) sustained in contact sports are thought to be necessary for the long-term development of chronic traumatic encephalopathy (CTE). Our objectives were to: 1) characterize the magnitude and persistence of RHI-induced white matter (WM) changes; 2) determine their relationship to kinematic measures of RHI; and 3) explore their clinical relevance.

**Methods:**

Prospective, observational study of 10 Division III college football players and 5 non-athlete controls during the 2011-12 season. All subjects underwent diffusion tensor imaging (DTI), physiologic, cognitive, and balance testing at pre-season (Time 1), post-season (Time 2), and after 6-months of no-contact rest (Time 3). Head impact measures were recorded using helmet-mounted accelerometers. The percentage of whole-brain WM voxels with significant changes in fractional anisotropy (FA) and mean diffusivity (MD) from Time 1 to 2, and Time 1 to 3 was determined for each subject and correlated to head impacts and clinical measures.

**Results:**

Total head impacts for the season ranged from 431–1,850. No athlete suffered a clinically evident concussion. Compared to controls, athletes experienced greater changes in FA and MD from Time 1 to 2 as well as Time 1 to 3; most differences at Time 2 persisted to Time 3. Among athletes, the percentage of voxels with decreased FA from Time 1 to 2 was positively correlated with several helmet impact measures. The persistence of WM changes from Time 1 to 3 was also associated with changes in serum ApoA1 and S100B autoantibodies. WM changes were not consistently associated with cognition or balance.

**Conclusions:**

A single football season of RHIs without clinically-evident concussion resulted in WM changes that correlated with multiple helmet impact measures and persisted following 6 months of no-contact rest. This lack of WM recovery could potentially contribute to cumulative WM changes with subsequent RHI exposures.

## Introduction

Although concussions are a frequent occurrence among athletes involved in contact sports such as American football, ice hockey, soccer, and lacrosse (1.6–3.8 million/year [1]), repetitive head impacts (RHI) that do not result in concussion are even more common. Using helmet-based accelerometers, estimates of the average number of RHIs in a single football season range from 244 to 1,444 per collegiate athlete [2], [3], and from 175 to 1,410 per high school athlete [2], [4]. In comparison, football-related concussion rates range from 64 to 76.8 per 100,000 athlete-exposures [5], [6], translating to approximately 0.05 concussions per athlete per season. Thus football players incur roughly 3,500–28,000 RHIs for every one concussion.

Several studies suggest that RHIs may be harmful to the brain in the short term. Head hits incurred during a boxing match without concussion have been associated with cognitive dysfunction [7] and acute brain injury [8]. Among high school athletes who did not experience concussion, RHIs during a single season of football were associated with abnormal regional cortical activation patterns on functional MRI [9], [10]. The magnitude of this activation correlated with the number of RHIs sustained during the season, and resembled that previously reported in subjects with frank concussion [11]. In a separate cohort, new learning on the California Verbal Learning Test declined over a single season of RHIs among collegiate football players who did not experience concussion [12]. Using helmet mounted accelerometers, poorer post-season reaction time and scores on the Trail Making test of visual attention and task switching were found to be associated with greater head impact exposures [12].

Evidence linking RHIs to longer-term brain problems such as chronic traumatic encephalopathy (CTE) is more tenuous. When traumatic neurodegeneration was first described among boxers in 1928 it was presumed that RHIs were responsible [13]. More recent autopsy series have detected CTE in other sports (American football, hockey, and wrestling [14], [15]), and also among individuals engaged in other activities such as repetitive head-banging [14]. In common to all of these cases was exposure to RHIs. Although frank concussions can occur with these activities, a pre-mortem history of concussion was absent in some of these cases [16], [17], raising the possibility that RHIs played a role in CTE [16], independent of concussion. Further, early-onset dementia has not been reported among athletes involved in sports such as rugby and Australian-rules football, where concussions are common but RHIs are not. (CTE has been reported in two rugby players but both also played American football [17]). Recognizing that RHIs play a key role in the development of CTE, but that not all athletes exposed to them develop CTE [17], [18], researchers at The Center for the Study of CTE at Boston University have concluded that repetitive brain trauma is “necessary but not sufficient,” for the development of CTE [19].

The mechanism by which RHIs might impair neurologic outcomes is not known. White matter (WM) changes detected on diffusion tensor imaging (DTI) after RHIs suggest a parallel to frank concussion. Reductions in fractional anisotropy (FA) [20], increases in mean diffusivity (MD) [20]–[23], and both [24] have been reported after RHIs in humans. These changes are thought to reflect traumatically-induced structural alterations in the neuronal axon and microenvironment [25]. However, the relationship of head impact forces and physiologic factors to these WM changes is not clear. Additionally it is unclear if these WM changes are transient or resolve with time.

To address these gaps, we performed DTI on a group of collegiate football players outfitted with helmet impact sensors prior to and after a single season of football, and then again after 6 months of no-contact rest. Our objectives were to: 1) quantify and characterize RHI-related WM changes at the end of the football season, and determine the persistence of these changes after a period of prolonged rest; 2) determine the relationship between kinematic measures of RHI and these WM changes; and 3) explore the clinical relevance of the observed WM changes in terms of cognitive function, balance, and select physiologic factors.

## Methods

We conducted a prospective study of 10 college football players and 5 non-athlete controls at the University of Rochester during the 2011 football season (August to December) and a subsequent 6-month no-contact rest period (December to May). Helmet impact measure data were collected from all athletes throughout the season using helmet-mounted accelerometers. WM changes and clinical correlates were assessed on each subject at the beginning of the football season (Time 1), at the end of the football season (Time 2), and after 6 months of rest from contact sports (Time 3). The University of Rochester Institutional Review Board approved this study and the process of informed consent; written informed consent was obtained from all participants.

### Subjects

Male athletes were recruited from the University of Rochester (UR) football team, which competes in National Collegiate Athletic Association Division III. Male controls were recruited from the UR general student body. Available resources and the novel nature of this investigation limited the maximum enrollment in this study to 15 subjects. Twice as many athletes as controls were chosen to maximize the power to detect significant correlations between helmet impact measures and WM changes. Ten active UR varsity football players were asked to participate and all agreed. These athletes were chosen for the variety of positions and anticipated head impacts they would experience during the season, which was informed by prior studies [3], [26]. Controls were selected based on response to a campus-wide call for research volunteers. Of the 10 student who responded, 5 were not eligible (4 had contraindications to MRI scanning, 1 played club rugby) and the remaining 5 were enrolled. Subjects, including controls, who were <18 years old or sustained a clinically diagnosed traumatic brain injury (TBI) of any severity within 2 weeks prior to the 2011 football season were excluded. History of prior TBI was determined by self-report using a previously validated survey tool [27].

### Primary Outcome: Changes in White Matter

Change in WM structure was the primary outcome and DTI was employed to measure these changes. This imaging modality is uniquely suited to detect the stretch-induced axonal damage thought to underlie all forms of TBI [28], [29]. This process results in the destruction of neurofilaments and microtubules spanning the length of the axon and leads to axonal swelling, followed by axonal disconnection and retraction. The linear arrangement of the axonal cytoskeleton is disrupted, as is the flow of water molecules down the axon [30]. These events are not detectable with computed tomography (CT) or conventional magnetic resonance imaging (MRI) [31]. DTI measures water movement in six or more non-collinear directions, allowing the determination of three mutually perpendicular eigenvalues, which coincide with the main water movement direction in white matter. Combinations of these three eigenvalues allow the derivations of two principle diffusion indices: fractional anisotropy (FA, represented by values ranging from 0 [random, multi-directional movement] to 1.0 [movement in one particular direction]), and mean diffusivity (MD, represented by a numerical value ranging from 0 [no movement] to 1.5×10^−2^ mm^2^/sec [totally unrestricted movement]).

For this study, DTI was acquired with a 3T Siemens Trio scanner using a single-shot pulsed-gradient SE-EPI sequence to measure FA and MD changes in WM. From the DTI images, voxel-wise comparisons of FA and MD were analyzed on a subject-specific basis. DTI data were analyzed using the wild bootstrapping permutation test, in which statistical significance of subject-specific voxel-wise changes in FA and MD were determined (described in detail elsewhere [24]). From this output, we calculated the percentage of all WM voxels with a statistically significant change (increase and/or decrease) in FA or MD from Time 1 to Time 2 and from Time 1 to Time 3 within each subject.

### Helmet Impact Measures

Each athlete was outfitted with a Riddell Revolution IQ helmet (Riddell Corporation; Elyria, OH) equipped with the Head Impact Telemetry System (HITS) encoders (Simbex LLC; Lebanon, NH) for the duration of the season, including all practices and games. These accelerometers record 40 milliseconds of data (8 milliseconds pre-trigger and 32 milliseconds post-trigger) at 1000 Hz for each head impact. Only impacts in which the calculated translational acceleration at the center of gravity of the player's head exceeded 10 g-forces (g) were recorded for analysis. Linear and rotational acceleration, the Gadd Severity Index (GSI), the Head Injury Criterion (HIC), and the Head Impact Technology suspect profile (HITsp) [2], were computed from the accelerometer data. The peak linear and peak rotational accelerations are the maximum magnitude of linear and rotational accelerations measured during an impact. The GSI and the HIC both measure a time integral of the linear acceleration to the 2.5 power but differ in the choice of time interval of integration [32]. The GSI is calculated by integrating over the full time of the impact, whereas the HIC is calculated by integrating only over the 15 milliseconds spanning the peak acceleration (and is thus commonly referred to as HIC15). The HIC is less likely than GSI to overestimate brain injury severity after low-intensity, long-duration impacts. Finally, the HITsp is a single metric computed from a principal component analysis and represents a weighted combination of peak linear acceleration, peak rotational acceleration, GSI, and HIC, along with information about impact location. This metric is an empirical metric that was shown to correlate with concussion in a previous study of football impacts [2].

### Clinical Measures

Subjects were also evaluated on the following clinical measures at all three study time points:

#### Cognitive performance

Cognitive performance was measured using the Immediate Post-Concussion Assessment and Cognitive Testing (ImPACT) test, a proprietary software program consisting of a concussion symptom inventory and six test modules measuring attention, memory and reaction time [33]. These modules are collectively used to generate three composite scores ranging from 0–100% (verbal memory, visual memory, visual motor speed), mean reaction time in seconds, and an impulse control score based on the sum of errors committed on two test modules (X's and 0's, color match). The cognitive efficiency index measures the interaction between accuracy and speed on one of the test modules (symbol match), with values ranging from zero to approximately 0.70. Finally, the concussion symptom inventory (CSI) is used to generate a post-concussive symptom score based on the frequency and severity of symptoms with total scores ranging from 0–132. These seven cognitive performance metrics were evaluated in each subject.

#### Balance

Postural stability was measured using the Balance Error Scoring System (BESS) and the Wii Balance Board (WBB). The BESS requires the subject to stand in three different stances (double leg, single leg, and in tandem) for 20 seconds with eyes closed and hands on hips [34]. Each stance was performed once on a firm surface and once on a 10-cm thick piece of medium-density foam. The BESS score is calculated by adding 1 error point for each performance error to a maximum of 60. The BESS has excellent intra-tester (0.88) and inter-tester (0.83) reliability [35]. The WBB was interfaced with a computer using custom-written software (Labview 8.5 National Instruments; Austin, TX, U.S.A.) while subjects performed 4 standing balance tasks: 1) single leg standing, eyes closed, 2) single leg standing, eyes open, 3) double leg standing, eyes closed and 4) double leg standing, eyes open. Data were collected for 10 seconds during single leg trials and for 30 seconds during double leg trials. The primary metric was center of pressure path length (cm) totaled for the 4 stances [36]. Longer path lengths indicate worse postural stability.

#### Physiologic Factors

Four milliliters of venous blood were drawn into sterile Vacutainer serum separator tubes and immediately placed on 0°C ice. Within 60 minutes, the blood was centrifuged (3000 rpm, 10 minutes), and the serum separated and stored at –80°C until sample analysis. Serum S100B concentrations were determined by ELISA manufactured by Diasorin (Stillwater, MN). 96 well plates were used and the analyte was sandwiched between two monoclonal antibodies directed against the beta-chain of the S100 dimer. Anti-human ELISA kits from Diasorin were read using a multi-plate fluorescent reader (at 450 nm). Fluorescent signals were converted into ng/mL as per standard curve concentrations. The detection limit of this ELISA is 0.01 ng/ml. The intra-assay coefficient of variance of this test is around 6%. S100B autoantibody and apoA-I concentrations were also measured in serum samples by ELISA. Maxisorp ELISA 96 wells plates were coated with a PBS solution containing S100B protein (human brain, catalog number-559291, EMD Chemicals), and serum apoA-I concentrations were measured in duplicate by ELISA (Mabtech; Cincinnati, OH). Apoε genotype was performed on DNA extracted from intra-oral cheek cells using the Hixson and Vernier method [37].

### Analysis

For each subject, the percentage of whole brain WM voxels with statistically significant changes from Time 1 to Time 2 and Time 1 to Time 3 were calculated for each of the of the following metrics: ↑FA, ↓FA, ↑MD, and ↓MD. Median percent changes were compared in athletes and controls using the Wilcoxon Rank Sum test. The percentage of WM voxels with significant interval changes in FA and MD was correlated to: 1) helmet impact metrics; and 2) clinical outcomes (including balance, cognitive performance, S100B, auto-S100B antibodies, and ApoA1 concentrations) using Spearman's correlation coefficient. Cognition and balance were correlated only to contemporaneous DTI changes, while physiologic variables were correlated to DTI changes occurring at all study time points. We posited that changes in clinical performance would be a result of WM changes and never preceded them. However, we anticipated that the physiologic milieu could not only be a result of WM changes (occurring contemporaneously or after) but could also influence the degree of WM changes (occurring before them).

Analyses were performed using SAS Software Version 9.3 (SAS Institute Inc.; Cary, NC, USA) and GraphPad Prism Version 5.02 for Windows (GraphPad Software; La Jolla, CA, USA). Statistical significance was defined as p<0.05, with p<0.10 also substantively important and interpreted as marginally significant given the small sample size. With a sample size of 10 athletes and 5 controls (assuming α = 0.05), we estimated approximately 90% power to detect a difference in the mean percentage of WM voxels with significant changes in FA between athletes and controls ranging from 0.6 to 0.8, and a difference in the mean percentage of WM voxels with significant changes in MD ranging from 0.80 to 1.0. Although wild bootstrapping includes an adjustment for multiple comparisons in brain voxels when calculating percentages of increased and decreased FA and MD, the correlation analysis linking DTI changes to head hits, serum biomarkers, and cognitive impairments was not adjusted for multiple comparisons. Rather, these correlations were presented graphically in a heat map table denoting tertile and direction of statistically significant r-values so that patterns could be more clearly discerned.

## Results

Baseline comparison to controls revealed that athletes had significantly higher body mass index and lower auto-S100B antibody titers, and lower impulse control (**Table 1**); athletes and controls were otherwise similar in the measured physiologic variables and clinical components. Two athletes and two controls were heterozygous for the ApoE4 allele. Only one athlete had a history of concussion (>2 weeks prior to study).

**Table 1:** Baseline comparison of athlete (n = 10) and control (n = 5) subjects.

	Athletes		Controls		
Parameter	Mean	(SD)	Mean	(SD)	*p*-value
Age (years)	20.4	(1.08)	20.6	(1.14)	0.81
Body Mass Index (kg/m^2^)	30.74	(1.58)	24.22	(2.02)	0.03
**Clinical Correlates**					
*Physiologic Measures*					
ApoE4 Positive, *n* (%)	2	(20.00)	2	(40.00)	0.56
ApoA1 (mg/dL)	122.5	(8.01)	149.2	(10.42)	0.07
S100B (ug/L)	0.107	(0.03)	0.059	(0.01)	0.34
S100B AutoAb Titer (Abs)	1.11	(0.27)	2.52	(0.06)	0.01
*Balance*					
Balance Error Scoring System	17.1	(4.70)	13.4	(5.30)	0.19
Center of Pressure Total Path Length (cm)	288.6	(33.00)	342.2	(132.00)	0.23
*Cognitive Performance*					
Visual Memory Score	74.3	(15.10)	70.2	(14.60)	0.63
Verbal Memory Score	90.4	(6.20)	85.6	(9.70)	0.26
Visual Motor Speed Score	47.2	(6.10)	43.8	(6.20)	0.33
Reaction Time (sec)	0.49	(0.05)	0.52	(0.04)	0.17
Impulse Control	3.0	(4.10)	6.6	(2.10)	0.04
Symptom	0.20	(0.63)	3.20	(5.20)	0.09
Cognitive Efficiency Score	0.45	(0.12)	0.39	(0.11)	0.40

### WM Changes

**Figure 1:**
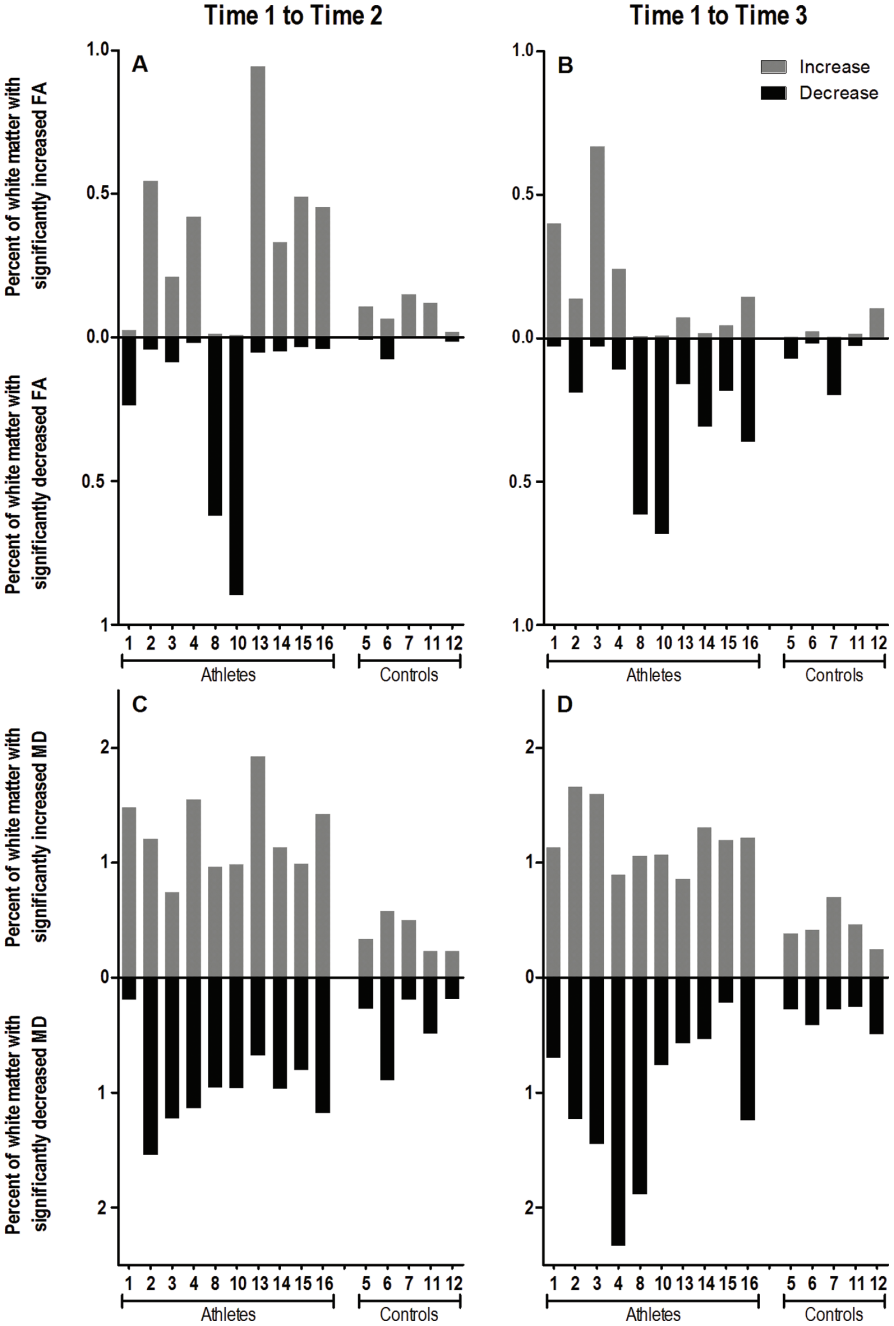
Subject-Specific DTI Changes. Subject-specific changes in FA (A and B) and MD (C and D) from Time 1 to Time 2 (A and C) and from Time 1 to Time 3 (B and D). Bars in each graph represent the percentage of white matter voxels in each individual subject with significantly decreased (black) and increased (grey) FA and MD over the specified time interval.

**Figure 2:**
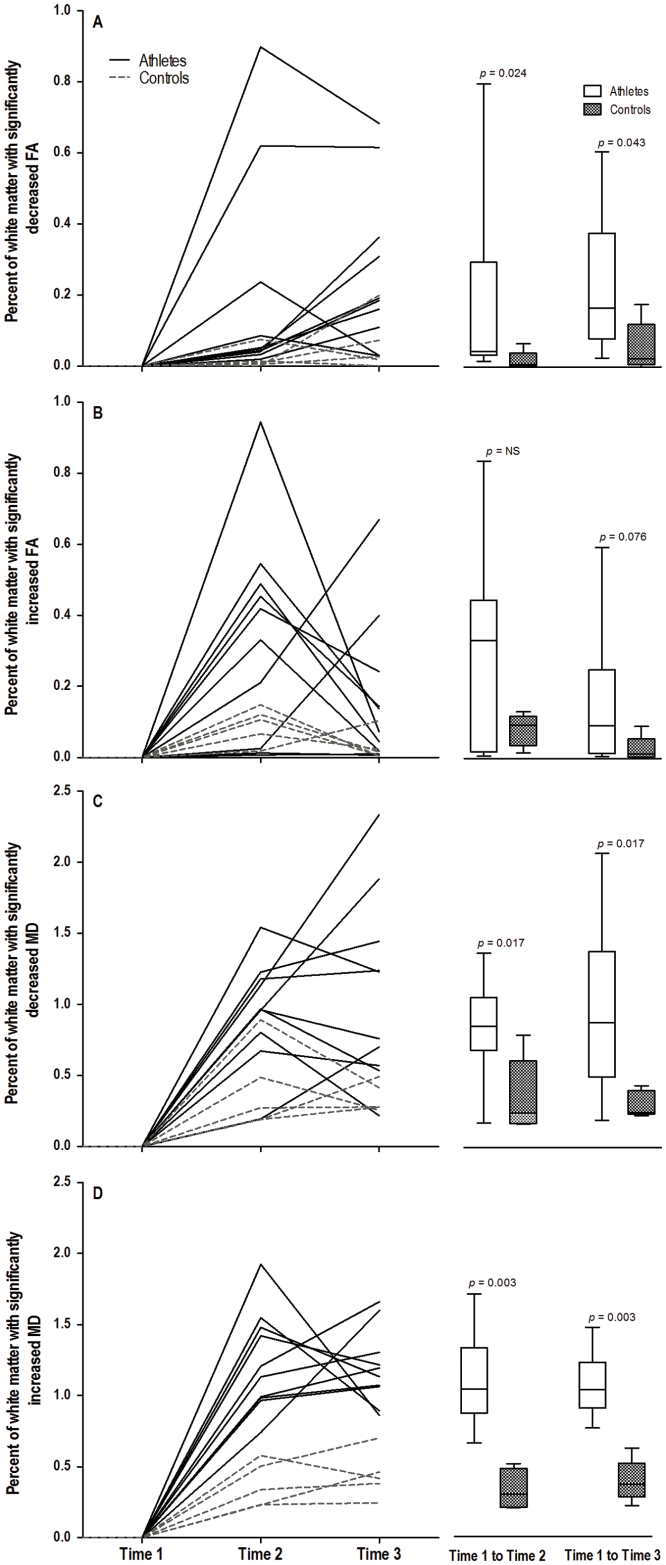
Trajectory of Subject-specific DTI Changes and Comparison by Subject Group. Line graphs show the percentage of WM voxels in each athlete (solid lines) and each control (hatched line) with significantly decreased (A and C) and increased (B and D) FA (A and B) and MD (C and D) from Time 1 to Time 2, and from Time 1 to Time 3. Box-and-whisker plots show the maximum and minimum (whiskers), inter quartile range (box) and median (line within box) values for the percentage of WM voxels in athletes (clear) and controls (black) with significantly decreased (A and C) and increased (B and D) FA (A and B) and MD (C and D) from baseline (Time 1) to 6 months of rest (Time 3).

**Figure 3:**
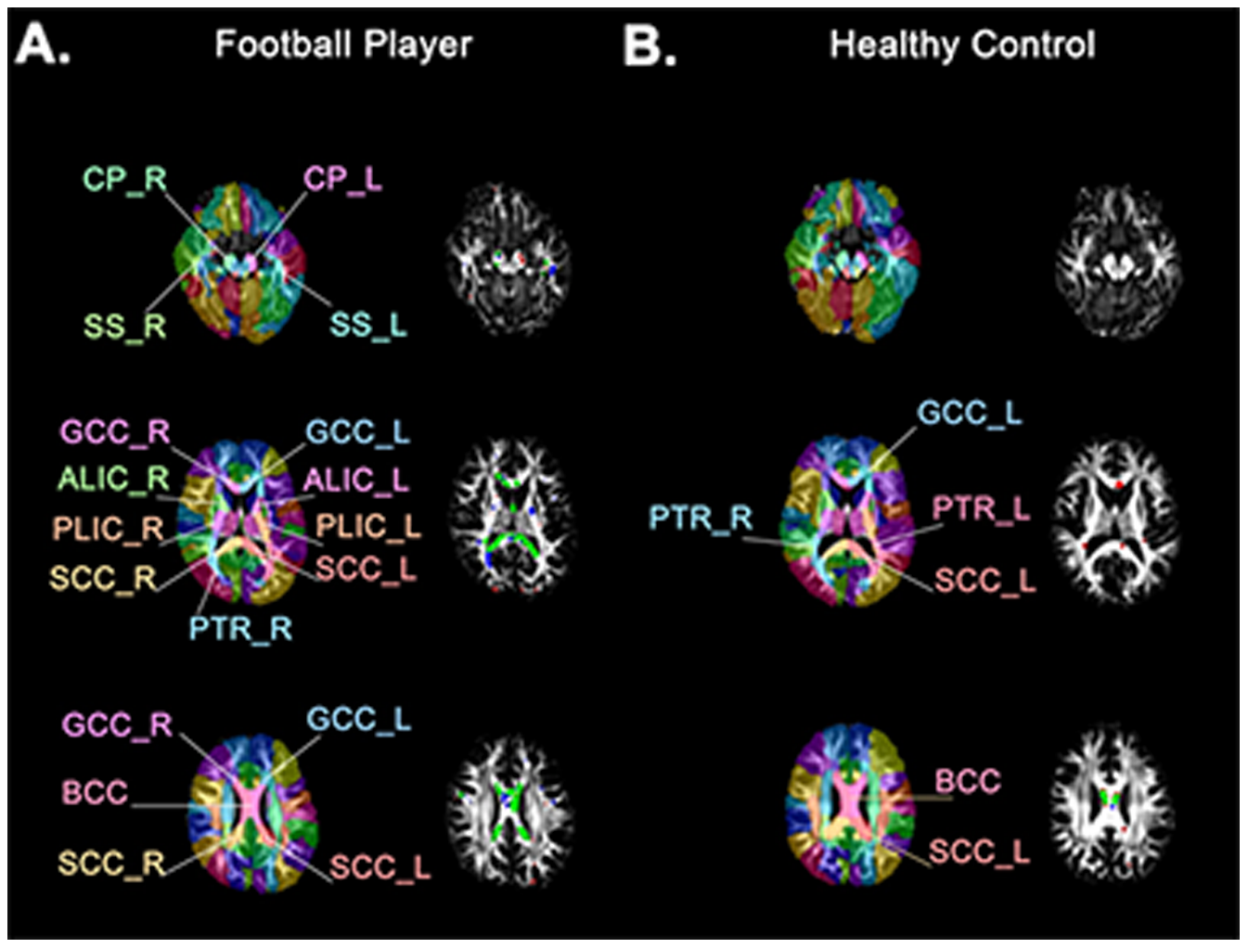
Spatial Distribution of WM Voxels with Decreased FA and Increased MD. WM structures (left), and significant DTI changes from Time 1 to Time 2 (right) in a football player (A) and a control subject (B). Columns 2 and 4 depict voxels with significant ↓FA (blue), significant ↑MD (red) and both ↓FA and ↑MD (green). ALIC: anterior limb of internal capsule; BCC: body of corpus callosum; CP: cerebral peduncle; GCC: genu of corpus callosum; PLIC: posterior limb of internal capsule; PTR: posterior thalamic radiation; SCC: splenium of corpus callosum; SS: sagittal stratum (includes inferior longitudinal fasciculus and inferior fronto-occipital fasciculus).

Compared to controls, athletes experienced greater WM changes in FA and MD from baseline (Time 1) to the end of the season (Time 2), as seen in **Figure 1, A and C**. These group differences were statistically significant for percentage of voxels with ↓FA (p = 0.024), ↓MD (p = 0.017), and ↑MD (p = 0.003), as shown in **Figure 2**. **↓**FA and ↑MD co-localized to the same brain voxels, especially in the corpus callosum (**Figure 3**).

Athletes also had greater voxel changes in FA and MD from baseline (Time 1) to the end of the 6-month no-contact rest period (Time 3), as seen in **Figure 1, panels B and D**. These group differences were statistically significant for ↓FA (p = 0.043), ↓MD (p = 0.017), and ↑MD (p = 0.003) (**Figure 2**). The difference in percentage of voxels with ↑FA was marginally significant (p = 0.076). Viewing the longitudinal trajectory in DTI changes in **Figure 2**, the significant differences observed at the end of the season (Time 2) in ↑MD, ↓MD and ↓FA persisted after 6 months of no-contact rest (Time 3).

### Helmet Impact Measures and Correlation with WM Change

Total head hits for the season ranged from 431 to 1,850. Summary helmet impact measures accrued during the 2011 football season for each athlete are shown in **Table 2**. Notably, none of the athletes suffered a clinically evident concussion during the study period.

**Table 2:** Helmet impact measures among athlete subjects (n = 10).

Position	Total Head Hits	Mean (SD) Linear Acceleration/Hit (g)		Mean (SD) Rotational Acceleration/Hit (rad/sec^2^)		Total Linear Acceleration (g)	Total Rotational Acceleration (rad/sec^2^)
Running Back	431	29.78	(20)	1880.53	(1513)	12,836.0	810,511
Tight End	572	31.41	(20)	1815.66	(1279)	18,033.0	1,042,191
Linebacker	612	37.53	(25)	1973.65	(1438)	22,969.3	1,207,879
Defensive Line	617	27.09	(14)	1704.77	(1034)	16,742.4	1,053,554
Defensive Line	649	28.23	(17)	1691.95	(1216)	18,325.1	1,098,081
Full Back	1,042	31.84	(22)	1877.13	(1572)	33,250.3	1,959,725
Linebacker	1,142	35.24	(24)	2071.71	(1578)	40,245.5	2,365,894
Defensive Line	1,423	34.36	(20)	2021.55	(1188)	48,903.4	2,876,675
Offensive Tackle	1,431	26.91	(14)	1726.87	(1026)	38,516.7	2,471,157
Center	1,850	31.88	(18)	1837.52	(1076)	58,994.1	3,399,421

**Figure 4:**
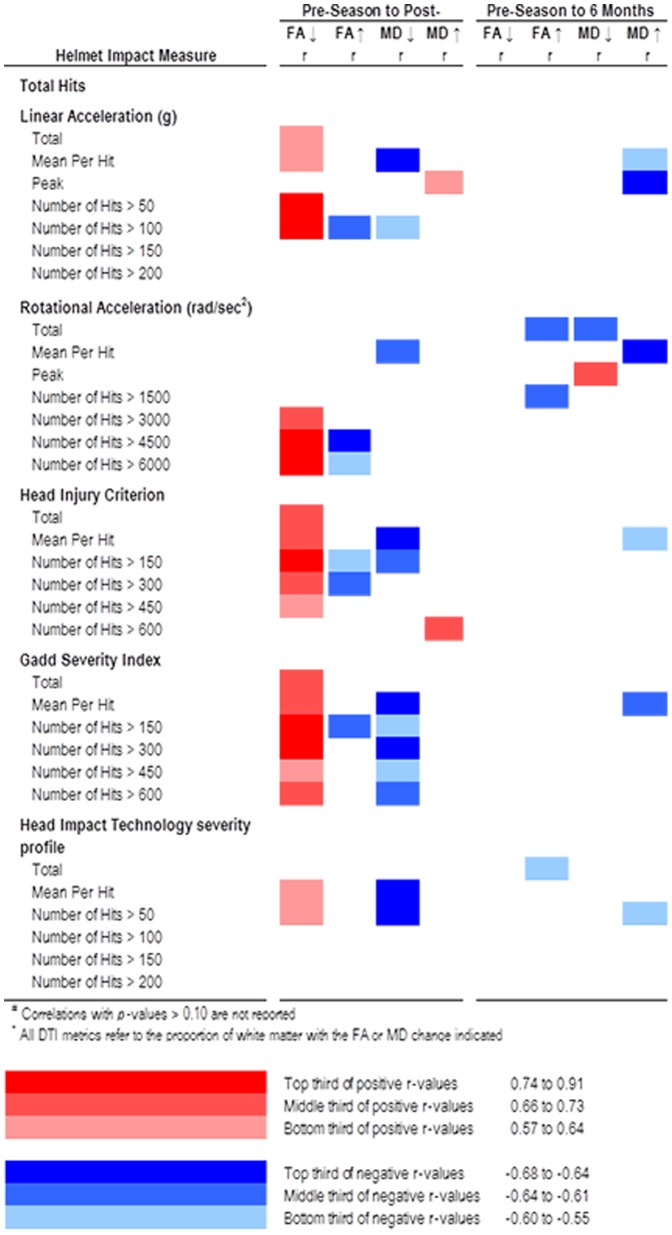
Heat map display of correlations between helmet impact measures and DTI changes between pre- and post-season (T1 to T2), and pre- and 6 months post-season (T1 to T3). Color and shading reflect direction and strength of correlation, as indicated by the figure key. Correlations with p-values>0.10 are not reported. All DTI metrics refer to the proportion of white matter with the FA or MD change indicated.

Among athletes, changes in FA and MD from Time 1 to Time 2 were associated with several helmet impact measures during the football season (**Figure 4**). Most of these significant correlations were related to the amount of FA decrease from Time 1 to Time 2. The impact measures with the most robust correlations were number of head hits with a peak rotational acceleration exceeding 4500 rad/sec^2^ (*r* = 0.91, p<0.001) and the number of head hits with a peak rotational acceleration exceeding 6000 rad/sec^2^ (*r* = 0.81, p<0.001). The direction of these correlations indicates that greater helmet impact measures were associated with greater percentage of voxels with *FA decrease*. The percentage of WM with decreased FA exceeded that of controls when the number of helmet impacts resulting in a peak rotational acceleration >4500 rads/sec^2^ exceeded 30–40 for the season, and when the number of helmet impacts resulting in a peak rotational acceleration >6000 rads/sec^2^ exceeded 10-15 for the season. A smaller number of helmet impact measures were significantly correlated to *MD decrease*. The direction of these correlations indicates that greater helmet impact measures were associated with a smaller percentage of voxels with ↓MD. DTI changes between Times 1 and 3 were not consistently associated with helmet impact measures.

### Clinical Correlates and WM Change

**Figure 5:**
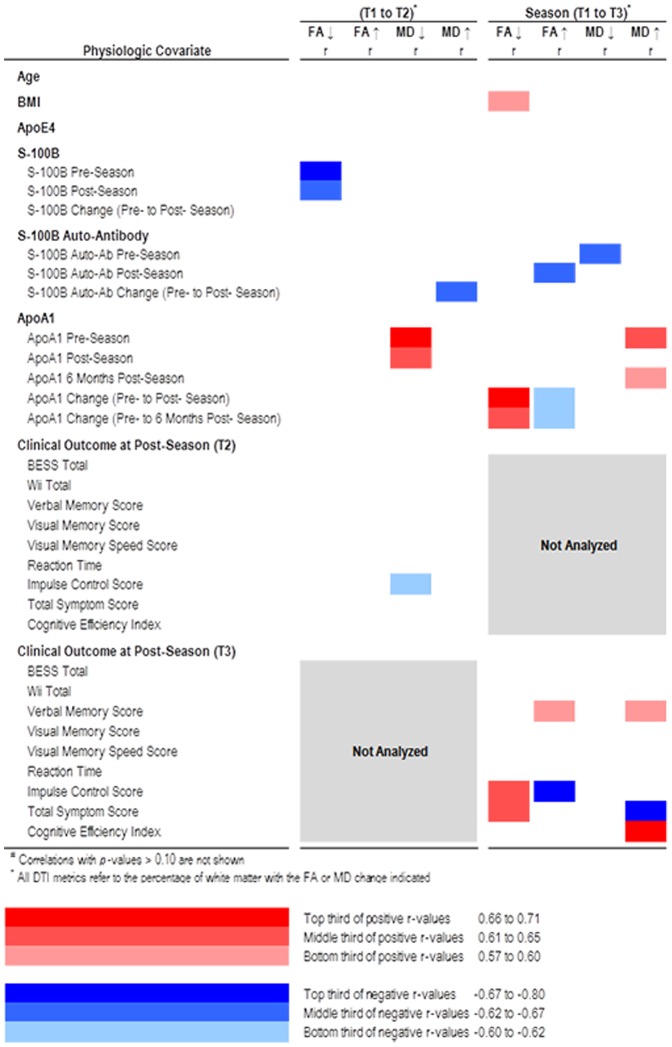
Physiologic and clinical correlations of DTI changes among athletes. Color and shading reflect direction and strength of correlation, as indicated by the figure key. Correlations with p-values>0.10 are not reported. All DTI metrics refer to the proportion of white matter with the FA or MD change indicated.

Several clinical correlates were also associated with Time 1 to Time 3 WM changes (**Figure 5**). Greater ↓FA was associated with an increase in serum ApoA1 from Time 1 to Time 2 (*r* = 0.661, p = 0.038) as well as from Time 1 to Time 3 (r = 0.648, p = 0.043). Greater ↑FA was associated with changes in ApoA1 in the opposite direction, that is, with a decrease from Time 1 to Time 2 (*r* = −0.612, p = 0.060), as well as from Time 1 to Time 3 (r = −0.612, p = 0.060). Greater ↑FA was also associated with lower S100B autoantibody titers at Time 2 (r = −0.624, p = 0.054). Greater ↓MD was associated lower S100B autoantibody titers at Time 1 (r = −0.673, p = 0.033). Greater ↑MD was associated with increased levels of serum ApoA1 at Time 1 (r = 0.612, p = 0.060) and Time 3 (r = 0.600, p = 0.067).

WM changes were not systematically associated with balance and cognitive performance measures among athletes at Time 2 or Time 3, but several patterns did emerge (**Figure 5**). DTI changes between Time 1 and Time 2 were not significantly correlated with most clinical outcome measures examined. However, DTI changes between Time 1 and Time 3 were correlated with both improved (↑FA, ↑MD) and worsened (↓FA) clinical outcomes as measured by cognitive performance.

## Discussion

In the current study we demonstrate that a single football season of RHIs resulted in significant changes in the structure of WM that persisted despite 6 months of no-contact rest. Post-season WM changes correlated with multiple head impact measures implying a potential causal relationship between helmet impact forces during a season of collegiate football and WM injury, despite no clinically evident concussion.

That these late, persistent changes in WM structure did not correlate with most clinical outcome measures suggests that they are for the most part clinically silent. This makes it difficult to establish with certainty whether these DTI changes are detrimental (i.e. represent damage or potential neuropathology) or in some way adaptive (i.e. represent neural plasticity). However, there were a few significant clinical correlations suggesting that some DTI changes may be detrimental while others are perhaps adaptive. For example, greater ↑FA between Time 1 and Time 3 was associated with improvements in verbal memory score and impulse control, while greater ↓FA was associated with worsening impulse control (**Figure 5**). Reciprocal changes in physiologic variables also support this concept. Greater ↑FA was associated with decreases in serum ApoA1 between Time 1 and Time 3 while greater ↓FA was associated with increases in serum ApoA1 (**Figure 5**). Although low serum ApoA1 levels are believed to increase the risk of dementia [38], [39], their relationship to post-TBI neurodegeneration has yet to be determined.

Others have also reported similar, sometimes clinically silent, DTI changes after non-concussive RHIs. Zhang et al compared 49 professional boxers to 19 healthy controls and found significantly increased mean MD and decreased mean FA in the corpus callosum and posterior limb of the internal capsule [20]. All boxers were considered free of neurologic disease although the methods used to make this determination were not described. Koerte et al compared pre- and postseason DTI scans among 17 male professional ice hockey players, three of whom experienced a clinically diagnosed concussion during the season [22]. Compared to preseason, postseason scans revealed significantly elevated trace, radial diffusion, and axial diffusion in the right precentral region, right corona radiata, and the anterior and posterior limbs of the internal capsule. Results were not given separately for the 14 players who did not suffer a concussion nor was their neurologic status. Additionally, Koerte et al compared the DTI scans of 12 male elite-level club soccer players to 8 male competitive swimmers, none of whom reported a history of concussion [21]. Soccer players had multiple brain regions with increased mean radial and axial diffusivity but none with significant changes in MD or FA. The neurologic status of the players was not reported. Our research group compared pre- and postseason DTI scans among nine high school football and hockey players (one of whom suffered a concussion during the season) and five healthy controls [24]. Among the eight athletes who did not suffer concussion, increases and decreases in both FA and MD were observed, but without significant changes in post-concussive symptoms or cognitive performance. Total changes in FA and MD among these athletes were over three times that of controls and also correlated to self-reported head hits during the season. Thus, while several studies have examined DTI in the setting of RHIs, few have related these changes to clinical outcomes.

Even fewer studies have linked DTI changes to head impact measures after RHI. However, McAllister et al compared DTI scans before and after a sport season in 80 non-concussed collegiate football and ice hockey players to 79 noncontact sport athletes. There was a significant athlete-group increase in MD in the corpus callosum. Postseason FA and MD in brain regions of interest were correlated with several helmet impact measures including total hits for the season, number of hits during the 14 days prior to scanning, seasonal 95th percentile rotational acceleration, HITsp, and linear acceleration [23].

If RHIs are related to neurodegeneration many years later, a long clinically silent period between the onset of neuronal injury and overt symptoms of dementia would not be unexpected. During this clinically silent period however, indicators of dysfunction on a cellular level are typically demonstrable. For example alterations in CSF levels of tau, phosphorylated tau, amyloid β_42_ and calbindin precede the development of overt symptoms of Alzheimer's disease [40], [41]. Our finding of altered ApoA1 and S100B autoantibodies in the serum may thus be analogous to these pre-AD changes in the CSF, potentially heralding the early stages of CTE. Pending confirmation in a long-term longitudinal study tracking athletes prospectively for years to decades looking for manifestations of early cognitive dysfunction and dementia, we believe our results suggest that these persistent DTI changes are likely detrimental.

If borne out in future research, the long-term persistence of these WM changes would mean that athletes returning to play the following season would be at risk for expanded RHI-related WM changes, undetectable by conventional assessments. Could the lack of WM recovery we observed result in cumulative WM damage with subsequent football seasons of RHI exposures? If so, could this cumulative WM damage be related to the long-term development of CTE? While we await confirmation for the long-term adverse effects of these WM changes, efforts to limit the development of RHI-related WM changes by monitoring helmet impact measures would seem prudent, and has already been suggested by The Sports Legacy Institute [42]. However rather than monitor total head hits, as has been suggested, it may be more effective to monitor those hits that are most likely to produce WM changes in excess of that seen among controls. In our relatively small sample, the percentage of WM with ↓FA exceeded that of controls when the number of helmet impacts resulting in a peak rotational acceleration >4500 rads/sec^2^ exceeded 30–40 for the season, and when the number of helmet impacts resulting in a peak rotational acceleration >6000 rads/sec^2^ exceeded 10–15 for the season.

There are several possible explanations for the long-term persistence of WM changes after a football season of RHIs. As a group, athletes did not show significant reduction in Time 1 to Time 2 WM changes during the six-month no-contact rest period. However, individually, some athletes demonstrated a return to baseline levels. Thus for some athletes, six months of rest may be sufficient for recovery, but for others more time may be necessary for postseason changes to return to baseline. In addition, our results suggest that changes in immunity may impact WM recovery. The significant correlations between S100B autoantibodies and WM changes suggest RHIs influence the immune response to repetitive antigen exposure. We have previously demonstrated that RHIs can result in intermittent low-level S100B release in football players [43]. Increased levels of serum S100B following in-season injury appears to lead to the elicitation of anti-S100B autoantibodies, and it is likely these athletes have encountered RHIs during prior seasons. As an innate and adaptive immune response, significant induction of regulatory T cells and other endogenous mechanisms that counteract autoimmunity and promote CNS repair during the off-season likely suppress the humoral response to this antigen, resulting in the lower autoantibody titers we observed among athletes as compared to controls at baseline (Time 1). Low Time 2 S100B autoantibody titers were associated with greater ↑FA between Time 1 and Time 3, while declines in S100B autoantibody titers between Time 1 and Time 2 were associated with greater ↑MD during that same time interval. Because the observed DTI changes were mostly clinically silent, the clinical implications of these associations are not clear. If the immune responses induced by RHIs contribute to the long-term neurodegeneration observed in those with CTE, as has been hypothesized [43], the mechanism(s) by which this adaptive response transitions to one that is maladaptive needs to be identified, and is a fruitful avenue for future research.

In the current study, we speculate that observed whole-brain DTI changes represent a mixture of cellular events. The seemingly paradoxical subject-specific concomitant increases and decreases in both FA and MD are likely due to the occurrence of multiple axonal injuries separated spatially in the brain and temporally across the 3 month football season. For any single athlete, multiple head impacts accrued over the course of the 3 month football season have the potential to injure more than one spatially-distinct area of brain. If injurious head impacts occur on different days, which they likely do, the time interval from injury to post-season scanning will vary for each injured brain area. Thus at the end of the football season each injured brain area will be in unique and possibly different stages in the evolution of traumatic axonal injury and/or repair. Thus the DTI scan of the whole brain done at the end of the football season likely reflects a combination of the cellular events occurring in the injured brain regions which may be at different axonal injury stages.

A limitation of our study is the use of non-athlete controls. The WM changes observed in athletes could have been due to physical exertion associated with college football in addition to brain injury. The clear separation observed between controls and athletes in the percentage of WM with ↑MD may be more likely to reflect the effect of physical, sports-related exertion on brain water diffusion rather than brain injury, as this DTI metric correlated with few helmet impact measures. Other DTI metrics, especially the percentage of WM with ↓FA, were more closely linked to helmet impact measures and thus may be less sensitive to the effects of exertion. Indeed, Gons et al reported significant changes in MD, but not FA, among 440 older adults reporting the highest levels of physical exercise [44]. Others have proposed the use of non-contact athletes as controls [21], but to our knowledge there has been no published reports demonstrating a significant difference in subject-specific DTI metrics between age-matched healthy non-athletes and non-contact athletes.

Additionally, although wild bootstrapping includes an adjustment for multiple voxel-wise comparisons, the analysis correlating DTI changes to head hits, antibodies, and cognitive impairments was not adjusted for multiple comparisons due to limited power afforded by the small sample size and the nascent state of the literature in this area. Regardless, many of the correlations were quite robust, with p-values <0.001. Furthermore, many of the observed correlations that were non-significant displayed moderate-to-large magnitude (r's>|0.50|).

Although one player admitted to having a prior concussion, there is no definitive method to exclude the possibility that any of the remaining players also had concussions prior to enrolling in the study. Our study was designed to specifically address this issue by comparing each athlete's postseason scan to their preseason scan. Thus, rather than evaluating absolute changes in WM structure, relative changes were examined. The effects on WM structure of a concussion occurring prior to beginning the study would not be expected to affect changes in WM structure from the beginning of the season to later time points. Finally, our ability to detect cognitive changes associated with a football season of RHIs may have been improved by the use of more sophisticated pencil-and-paper cognitive testing, rather than the computerized format afforded by ImPACT. For example, McAllister et al detected cognitive changes after a single football season of RHIs by using the California verbal learning test^12^. It may also have been improved by following subjects beyond 6 months for the development of delayed post concussive symptoms or cognitive deficits. The relationship between RHI-associated WM changes and more subtle cognitive changes is yet another fertile area for future research.

Collectively, in this preliminary study, we have demonstrated that a single football season of RHIs without clinically evident concussion resulted in WM changes on DTI. These DTI changes correlated with multiple helmet impact measures and persisted despite 6 months of no-contact rest. This lack of WM recovery could potentially contribute to progressive, cumulative WM damage with subsequent RHI exposures. If this relationship is confirmed in longitudinal studies, efforts to limit the development of RHI-related WM changes by monitoring helmet impact measures, and further elucidation of modifiable factors that may influence WM recovery, could mitigate the long-term risk of CTE.

## References

[1] Langlois JA, Rutland-Brown W, Wald MM (2006) The epidemiology and impact of traumatic brain injury: a brief overview. Journal of Head Trauma Rehabilitation 21: 375–378. 16983222 10.1097/00001199-200609000-00001

[2] Greenwald RM, Gwin JT, Chu JJ, Crisco JJ, Greenwald RM, et al (2008) Head impact severity measures for evaluating mild traumatic brain injury risk exposure. Neurosurgery 62: 789–798 discussion 798. 18496184 10.1227/01.neu.0000318162.67472.ad PMC2790598

[3] Crisco JJ, Fiore R, Beckwith JG, Chu JJ, Brolinson PG, et al (2010) Frequency and location of head impact exposures in individual collegiate football players. Journal of Athletic Training 45: 549–559. 21062178 10.4085/1062-6050-45.6.549 PMC2978006

[4] Broglio SP, Schnebel B, Sosnoff JJ, Shin S, Fend X, et al (2010) Biomechanical properties of concussions in high school football. Medicine & Science in Sports & Exercise 42: 2064–2071. 20351593 10.1249/MSS.0b013e3181dd9156 PMC2943536

[5] Marar M, McIlvain N, Fields S, Comstock D (2012) Epidemiology of Concussions Among United States High School Athletes in 20 Sports. The American Journal of Sports Medicine 40: 747–755. 22287642 10.1177/0363546511435626

[6] Meehan WP 3rd, d'Hemecourt P, Collins CL, Comstock RD (2011) Assessment and management of sport-related concussions in United States high schools. Am J Sports Med 39: 2304–2310. 21969181 10.1177/0363546511423503 PMC3359792

[7] McCrory P, Zazryn T, Cameron P, McCrory P, Zazryn T, et al (2007) The evidence for chronic traumatic encephalopathy in boxing. Sports Medicine 37: 467–476. 17503873 10.2165/00007256-200737060-00001

[8] Zetterberg H, Hietala MA, Jonsson M, Andreasen N, Styrud E, et al (2006) Neurochemical aftermath of amateur boxing. Archives of Neurology 63: 1277–1280. 16966505 10.1001/archneur.63.9.1277

[9] Talavage TM, Nauman EA, Breedlove EL, Yoruk U, Dye AE, et al. (2010) Functionally-Detected Cognitive Impairment in High School Football Players Without Clinically-Diagnosed Concussion. Journal of Neurotrauma doi:10.1089/neu.2010.1512. 10.1089/neu.2010.1512 PMC3922228 20883154

[10] Breedlove EL, Robinson M, Talavage TM, Morigaki KE, Yoruk U, et al (2012) Biomechanical correlates of symptomatic and asymptomatic neurophysiological impairment in high school football. Journal of Biomechanics 45: 1265–1272. 22381736 10.1016/j.jbiomech.2012.01.034

[11] Jantzen KJ (2010) Functional magnetic resonance imaging of mild traumatic brain injury. Journal of Head Trauma Rehabilitation 25: 256–266. 20611044 10.1097/HTR.0b013e3181e5477c

[12] McAllister TW, Flashman LA, Maerlender A, Greenwald RM, Beckwith JG, et al (2012) Cognitive effects of one season of head impacts in a cohort of collegiate contact sport athletes. Neurology 78: 1777–1784. 22592370 10.1212/WNL.0b013e3182582fe7 PMC3359587

[13] Martland HS (1928) Punch Drunk. JAMA 91: 1103–1107.

[14] McKee AC, Cantu RC, Nowinski CJ, Hedley-Whyte ET, Gavett BE, et al (2009) Chronic traumatic encephalopathy in athletes: progressive tauopathy after repetitive head injury. Journal of Neuropathology & Experimental Neurology 68: 709–735. 19535999 10.1097/NEN.0b013e3181a9d503 PMC2945234

[15] Omalu B, Bailes J, Hamilton RL, Kamboh MI, Hammers J, et al (2011) Emerging histomorphologic phenotypes of chronic traumatic encephalopathy in American athletes. Neurosurgery 69: 173–183 discussion 183. 21358359 10.1227/NEU.0b013e318212bc7b

[16] Baugh CM, Stamm JM, Riley DO, Gavett BE, Shenton ME, et al (2012) Chronic traumatic encephalopathy: neurodegeneration following repetitive concussive and subconcussive brain trauma. Brain Imaging & Behavior 6: 244–254. 22552850 10.1007/s11682-012-9164-5

[17] McKee AC, Stein TD, Nowinski CJ, Stern RA, Daneshvar DH, et al (2012) The spectrum of disease in chronic traumatic encephalopathy. Brain 136: 43–64. 23208308 10.1093/brain/aws307 PMC3624697

[18] Hazrati L-N TM, Diamandis P, Davis KD, Green RE, Wennberg R WJ, Ezerins L, Tator CH (2013) Absence of chronic traumatic encephalopathy in retired football players with multiple concussions and neurological symptomatology. Frontiers in Human Neuoscience 2: 1–9. 10.3389/fnhum.2013.00222 PMC3662898 23745112

[19] Stern RA, Riley DO, Daneshvar DH, Nowinski CJ, Cantu RC, et al (2011) Long-term consequences of repetitive brain trauma: chronic traumatic encephalopathy. Pm & R 3: S460–467. 22035690 10.1016/j.pmrj.2011.08.008

[20] Zhang L, Heier LA, Zimmerman RD, Jordan B, Ulug AM (2006) Diffusion anisotropy changes in the brains of professional boxers. Ajnr: American Journal of Neuroradiology 27: 2000–2004. 17032883 PMC7977918

[21] Koerte IK, Ertl-Wagner B, Reiser M, Zafonte R, Shenton ME (2012) White matter integrity in the brains of professional soccer players without a symptomatic concussion. JAMA 308: 1859–1861. 23150002 10.1001/jama.2012.13735 PMC4103415

[22] Koerte IK KD, Hartl E, Bouix S, Pasternak O, Kubicki M, Rauscher A, Li D, Dadachanji SB, Taunton JA, Forwell LA, Johnson AM, Echlin PS, Shenton ME (2012) A prospective study of physician-observed concussion during a varsity university hockey season: white matter integrity in ice hockey players. Part 3 of 4. Neurosugical Focus 33: E3. 10.3171/2012.10.FOCUS12303 PMC5687247 23199426

[23] McAllister TW FJ, Flashman LA, Maerlender A, Greenwald RM, Beckwith JG, Bolander RP, Tosteson TD, Turcl JH, Raman R, Jain S. (2013) Effect of head impacts on diffusivity measures in a cohort of collegiate contact sport athletes. Neurology DOI10.1212/01.wnl.0000438220.16190.42. 10.1212/01.wnl.0000438220.16190.42 PMC3873621 24336143

[24] Bazarian JJ, Zhu T, Blyth B, Borrino A, Zhong J (2012) Subject-specific changes in brain white matter on diffusion tensor imaging after sports-related concussion. Magnetic Resonance Imaging 30: 171–180. 22079073 10.1016/j.mri.2011.10.001 PMC3254806

[25] Fujita M, Wei EP, Povlishock JT (2012) Intensity- and interval-specific repetitive traumatic brain injury can evoke both axonal and microvascular damage. Journal of Neurotrauma 29: 2172–2180. 22559115 10.1089/neu.2012.2357 PMC3419839

[26] Mihalik JP, Bell DR, Marshall SW, Guskiewicz KM (2007) Measurement of head impacts in collegiate football players: an investigation of positional and event-type differences. Neurosurgery 61: 1229–1235 discussion 1235. 18162902 10.1227/01.neu.0000306101.83882.c8

[27] Corrigan JD, Bogner J (2007) Initial reliability and validity of the Ohio State University TBI Identification Method. Journal of Head Trauma Rehabilitation 22: 318–329. 18025964 10.1097/01.HTR.0000300227.67748.77

[28] Blumbergs PC, Scott G, Manavis J, Wainwright H, Simpson DA, et al (1995) Topography of axonal injury as defined by amyloid precursor protein and the sector scoring method in mild and severe closed head injury. J Neurotrauma 12: 565–572. 8683607 10.1089/neu.1995.12.565

[29] Blumbergs PC, Scott G, Manavis J, Wainwright H, Simpson DA, et al (1994) Staining of amyloid precursor protein to study axonal damage in mild head injury. Lancet 344: 1055–1056. 7523810 10.1016/s0140-6736(94)91712-4

[30] Buki A, Povlishock JT (2006) All roads lead to disconnection?—Traumatic axonal injury revisited. Acta Neurochirurgica 148: 181–193 discussion 193–184. 16362181 10.1007/s00701-005-0674-4

[31] Bazarian JJ, Blyth B, Cimpello L (2006) Bench to bedside: evidence for brain injury after concussion—looking beyond the computed tomography scan. Academic Emergency Medicine 13: 199–214. 16436787 10.1197/j.aem.2005.07.031

[32] Hayes WC, Erickson MS, Power ED (2007) Forensic injury biomechanics. Annual Review of Biomedical Engineering 9: 55–86. 10.1146/annurev.bioeng.9.060906.151946 17447861

[33] Collins MW, Iverson GL, Lovell MR, McKeag DB, Norwig J, et al (2003) On-field predictors of neuropsychological and symptom deficit following sports-related concussion. Clinical Journal of Sport Medicine 13: 222–229. 12855924 10.1097/00042752-200307000-00005

[34] Riemann B, Guskiewicz K (1997) Assessment of mild head injury using measures of balance and cognition: a case study. Journal of Sport Rehabilitation 6: 283–289.

[35] Finnoff JT, Peterson VJ, Hollman JH, Smith J, Finnoff JT, et al (2009) Intrarater and interrater reliability of the Balance Error Scoring System (BESS). Pm & R 1: 50–54. 19627872 10.1016/j.pmrj.2008.06.002

[36] Clark RA, Bryant AL, Pua Y, McCrory P, Bennell K, et al (2010) Validity and reliability of the Nintendo Wii Balance Board for assessment of standing balance. Gait & Posture 31: 307–310. 20005112 10.1016/j.gaitpost.2009.11.012

[37] Hixson JE, Vernier DT (1990) Restriction isotyping of human apolipoprotein E by gene amplification and cleavage with HhaI. Journal of Lipid Research 31: 545–548. 2341813

[38] Saczynski JS, White L, Peila RL, Rodriguez BL, Launer LJ (2007) The relation between apolipoprotein A-I and dementia: the Honolulu-Asia aging study. American Journal of Epidemiology 165: 985–992. 17298957 10.1093/aje/kwm027

[39] Merched A, Xia Y, Visvikis S, Serot JM, Siest G (2000) Decreased high-density lipoprotein cholesterol and serum apolipoprotein AI concentrations are highly correlated with the severity of Alzheimer's disease. Neurobiology of Aging 21: 27–30. 10794845 10.1016/s0197-4580(99)00103-7

[40] Craig-Schapiro R, Kuhn M, Xiong C, Pickering EH, Liu J, et al (2011) Multiplexed immunoassay panel identifies novel CSF biomarkers for Alzheimer's disease diagnosis and prognosis. PLoS ONE [Electronic Resource] 6: e18850. 10.1371/journal.pone.0018850 PMC3079734 21526197

[41] Perrin RJ, Fagan AM, Holtzman DM (2009) Multimodal techniques for diagnosis and prognosis of Alzheimer's disease. Nature 461: 916–922. 19829371 10.1038/nature08538 PMC2810658

[42] Cantu R, Nowinski C (February 2012) Sports Legacy Institute, Hit Count Initiative. http://wwwsportslegacyorg/policy/hit-count/ Last accessed Nov. 24, 2013.

[43] Marchi N, Bazarian J, Puvenna V, Janigro M, Ghosh C, et al (2013) Consequences of Repeated Blood-Brain Barrier Disruption in Football Players. PLoS ONE [Electronic Resource] 8: e56805 doi:56810.51371/journal.pone.0056805 10.1371/journal.pone.0056805 PMC3590196 23483891

[44] Gons RAR, Tuladhar AM, de Laat KF, van Norden AGW, van Dijk EJ, et al (2013) Physical activity is related to the structural integrity of cerebral white matter. Neurology 81: 971–976. 23921884 10.1212/WNL.0b013e3182a43e33

